# EGF‐Induced Macropinocytosis Promotes NAV1‐Dependent Internalization of Occludin in Keratinocytes

**DOI:** 10.1096/fj.202402876R

**Published:** 2025-04-23

**Authors:** Haruka Taira, Lixin Li, Asumi Koyama, Rino Toyoshima, Toyoki Yamamoto, Yukiko Ito, Eiki Sugimoto, Yuka Mizuno, Kentaro Awaji, Shinichi Sato, Sayaka Shibata

**Affiliations:** ^1^ Department of Dermatology, Graduate School of Medicine The University of Tokyo Tokyo Japan

**Keywords:** atopic, dermatitis, endocytosis, rho GTP‐binding proteins, tight junction proteins

## Abstract

Epidermal keratinocytes form the outermost layer of the skin and serve as a pivotal barrier against external insults. This barrier, however, can be compromised in conditions such as atopic dermatitis (AD), where both genetic and environmental factors contribute to its disruption. Recent studies have indicated that macropinocytosis, a non‐selective endocytic process, is involved in the internalization of barrier proteins. In this study, we explored the role of macropinocytosis in differentiated keratinocytes and its potential impact on skin barrier integrity in AD. Our results demonstrated that epidermal growth factor (EGF), but not the type 2 cytokines IL‐4 and IL‐13, significantly promoted macropinocytosis in differentiated HaCaT keratinocytes. EGF stimulation increased the uptake of 70 kDa dextran and induced the internalization of occludin, a component of tight junction proteins. Furthermore, enhanced macropinocytosis was observed in the epidermis of a mouse model of AD, accompanied by elevated EGF expression in the skin, indicating that the AD skin microenvironment may drive this process. NAV1 was identified as a critical regulator of EGF‐induced macropinocytosis, as its knockdown significantly impaired this process. Transcriptome analysis of NAV1‐knockdown cells further revealed changes in the expression of Rho family GTPases, including *CDC42* and *MMP14*, suggesting that NAV1 modulates macropinocytosis through Rho‐dependent pathways. These findings provide new insights into the regulation of macropinocytosis in keratinocytes and its potential contribution to the barrier dysfunction observed in AD.

## Graphical abstract

This graphical abstract illustrates how epidermal growth factor (EGF) stimulates macropinocytosis in keratinocytes, leading to the internalization of occludin, a key tight junction protein. NAV1 is identified as a critical mediator of this process, modulating macropinocytosis via the Rho family of GTPases. These findings suggest that EGF‐induced macropinocytosis may contribute to barrier dysfunction in inflammatory skin diseases such as atopic dermatitis.

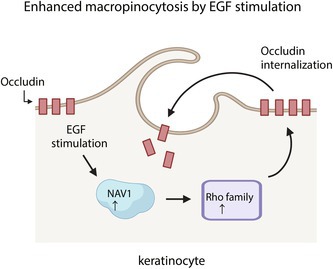

## 1 Introduction

Atopic dermatitis (AD) is a chronic inflammatory skin disorder that typically begins in infancy and severely impacts the quality of life of those affected [1]. The pathophysiology of AD is multifaceted, with genetic factors like filaggrin gene mutations playing a critical role in compromising the skin's barrier function [2]. This impaired barrier allows external antigens to penetrate the skin, triggering immune responses dominated by type 2 inflammation through cytokines such as IL‐4 and IL‐13 [3]. These cytokines further suppress the expression of filaggrin and tight junction (TJ) proteins, leading to a cycle of ongoing barrier disruption and immune activation [3]. This vicious cycle not only exacerbates the disease but also increases the skin's susceptibility to environmental allergens, perpetuating a feedback loop that deepens the pathology of AD [4].

While the role of type 2 inflammation in AD is well‐established, new techniques, including single‐cell analysis, have revealed that pathways linked to type 1 (IFN‐γ) and type 3 (IL‐17A, IL‐17F) responses are also activated in AD skin lesions [5, 6]. Additionally, growth factors such as EGF and TGF‐α, which are involved in various cellular processes, including the regulation of keratinocyte and fibroblast activity, further contribute to the disease's complexity [7, 8]. The role of these alternative pathways in barrier dysfunction is less understood, indicating a need for further research into these mechanisms.

Macropinocytosis, a non‐selective endocytic process, is integral to various cellular functions, including nutrient uptake in cancer cells and antigen presentation in immune cells like dendritic cells and macrophages [9, 10]. During macropinocytosis, cells engulf extracellular fluid and its contents, including proteins and antigens, into large vesicles called macropinosomes [11, 12]. In epithelial tissues, this process has been associated with the internalization of tight junction components, potentially compromising barrier integrity [13]. Indeed, excessive endocytosis triggered by type 1 inflammation and growth factors in the intestinal epithelium has been associated with barrier dysfunction [13, 14, 15].

In the epidermis, macropinocytosis has broader implications beyond nutrient uptake, playing roles in various physiological and pathological processes. It has been associated with the internalization of type XVII collagen, which contributes to the pathogenesis of bullous pemphigoid (BP) [16, 17]. Furthermore, macropinocytosis facilitates melanosome uptake during the transfer of melanin from melanocytes to keratinocytes, indicating its involvement in pigmentation mechanisms [18]. Although these processes have been observed in undifferentiated keratinocytes, the function of macropinocytosis in maintaining the epidermal barrier, particularly within differentiated keratinocytes, remains underexplored. Given the involvement of various inflammatory pathways and growth factors in AD, it is plausible that macropinocytosis may also involve the breakdown of the epidermal barrier by promoting the internalization of tight junction proteins.

This study aims to clarify the mechanisms by which macropinocytosis induces the breakdown of epidermal barrier integrity in keratinocytes. By exploring the role of macropinocytosis in this context, we aim to uncover new insights into the regulation of skin barrier function and identify potential strategies for therapeutic intervention in AD.

## 2 Materials and Methods

### 2.1 Cell Culture of HaCaT Cells

HaCaT cells, a spontaneously immortalized human keratinocyte line, were provided by Dr. Komine (Jichi Medical School, Tochigi, Japan). The cells were cultured in Eagle's minimum essential medium (MEM) from Sigma‐Aldrich (St Louis, MO) containing 10% fetal bovine serum (Gibco, Grand Island, NY) and antibiotics in 5% CO_2_ at 37°C. Cells were treated with a final concentration of 2.8 mM calcium chloride in MEM for 4 days to induce differentiation.

### 2.2 Mice and MC903‐Induced AD‐Like Skin Dermatitis Model

C57BL/6 mice were purchased from SLC Japan (Shizuoka, Japan) and maintained under specific pathogen‐free conditions with a 12‐h light/dark cycle and free access to food and water. Mice aged 8–11 weeks of either sex were used for the experiments. These mice were randomly divided into two groups: the mock‐treated group (vehicle‐treated control) and the MC903‐treated group. To induce AD‐like skin dermatitis, 20 μL of 2 nM MC903 (Tocris Bioscience, Bristol, UK) dissolved in ethanol was applied to the ears daily for nine consecutive days. The mock‐treated group received 20 μL of ethanol (vehicle) alone under the same conditions. Ear thickness was measured daily using a dial thickness gauge (4‐3086‐03; As One Corporation, Tokyo, Japan). No inclusion or exclusion criteria were set for these mouse experiments. HT was responsible for the topical application and was the sole researcher with knowledge of the group allocation. The measurement of ear thickness was conducted by HT and SS, with the latter being blinded to the group assignments. All studies and procedures were approved by the Committee on Animal Experimentation of the University of Tokyo Graduate School of Medicine. All animal experiment procedures and animal care were conducted according to the ARRIVE guidelines [19].

### 2.3 Human Samples From Healthy Controls and Patients With AD

Skin samples were collected from 4 healthy controls and 4 AD patients after obtaining written informed consent. Samples of AD patients were taken from the lesional skin that had not received systemic treatment. The medical ethics committee of the University of Tokyo approved all described studies (No. 0695), and the study was conducted according to the principles of the Declaration of Helsinki.

### 2.4 Assessment of Macropinocytosis by Flow Cytometry in HaCaT Cells and MC903‐Induced AD‐Like Skin Dermatitis Model

HaCaT cells were seeded into a 24‐well plate, and 4 days after differentiation, cells were stimulated with EGF (10 ng/mL, R&D Systems) or with cytokines IL‐4 (100 ng/mL, Peprotech, Rocky Hill, NJ) and IL‐13 (100 ng/mL, Peprotech), or left untreated. To evaluate macropinocytosis, 70 kDa FITC‐dextran (Sigma‐Aldrich) was added to a final concentration of 0.5 mg/mL, and the cells were incubated at 37°C for 6 h. In some experiments, cells were pretreated with 50 or 100 μM ethylisopropylamiloride (EIPA, Sigma‐Aldrich) 1 h before the stimulation with 70 kDa FITC‐dextran to assess macropinocytosis specificity. In certain experiments, HaCaT cells were cultured under two conditions to assess the impact of differentiation: (1) differentiated (2.8 mM, 4 days in high calcium medium) or (2) undifferentiated (4 days in standard low calcium medium). To assess ligand specificity, TGF‐α (10 ng/mL) was used. After incubation, cells were then detached with trypsin–EDTA (Lonza, Basel, Switzerland) to obtain a single‐cell suspension for further analysis.

For the MC903‐induced AD‐like skin dermatitis model, on the day after the last MC903 application (Day 10), 50 μL of 70 kDa FITC‐dextran (5 mg/mL) was applied to the inner ears of mice. After 6 h, mice were sacrificed, and the collected ears were split into inner and outer sides. The tissues were floated on the surface of 0.25% trypsin (Gibco) at 4°C overnight to isolate a single‐cell suspension of epidermal cells. In some experiments, EIPA was applied 1 h before the application of MC903 on Days 5–9. After obtaining the single‐cell suspension, cells were fixed with 2% paraformaldehyde at room temperature for 10 min. Live cells were identified with Zombie NIR fluorescent dye (BioLegend, San Diego, CA).

To quantitatively assess macropinocytosis, the median fluorescence intensity (MFI) of FITC‐positive cells was analyzed using a CytoFLEX flow cytometer (Beckman Coulter, Brea, CA).

### 2.5 Immunofluorescence Staining

HaCaT cells were seeded on chamber slides (Matsunami Glass Ind, Osaka, Japan), and 4 days after differentiation, cells were treated with or without the indicated concentrations of EGF or IL‐4/IL‐13. EIPA (10 μM) was added 1 h before EGF stimulation for macropinocytosis assessment. After fixation, blocking was conducted with 5% Bovine Serum Albumin (BSA) in PBS. Cells were then incubated with the primary antibody against occludin (Invitrogen, Carlsbad, CA) overnight at 4°C, followed by the secondary antibody, Alexa Fluor 594‐conjugated anti‐mouse antibody (Jackson ImmunoResearch Inc., West Grove, PA). Immunofluorescence images were captured using the all‐in‐one fluorescence microscope BZ‐X810 (Keyence, Itasca, IL).

### 2.6 Immunohistochemistry Staining

Formalin‐fixed paraffin‐embedded skin sections were deparaffinized, permeabilized with 0.2% Triton‐X100 in PBS, and underwent heat‐induced antigen retrieval with a citrate‐based antigen unmasking solution (Vector Laboratories, Burlingame, CA). After blocking, sections were incubated with primary antibodies against mouse NAV1 (Proteintech) or human NAV1 (Atlas Antibodies, Stockholm, Sweden) overnight at 4°C. Slides were then treated with biotin‐conjugated secondary antibodies (Vector Laboratories), followed by Vectastain Elite ABC reagents, and stained with 3,3′‐Diaminobenzidine Tetrahydrochloride (Vector Laboratories). NAV1 staining for human sections was quantified using the BZ‐X810 microscope and BZ‐X Analyzer software (Keyence), as previously described [20]. Briefly, different threshold values were set for the epidermis and dermis, and the percentage of the stained area exceeding these thresholds was automatically calculated.

### 2.7 SDS‐Polyacrylamide Gel Electrophoresis and Immunoblotting

Proteins from HaCaT cells were extracted using Radio‐Immunoprecipitation Assay lysis buffer (Santa Cruz Biotechnology, Santa Cruz, CA). Protein samples were separated by sodium dodecyl sulfate (SDS)‐polyacrylamide gel electrophoresis and transferred to the Immobilon‐P transfer membrane (Millipore, Bedford, MA). The membrane was blocked in 5% non‐fat milk buffer and incubated with primary antibodies against occludin (Invitrogen) or β‐actin (CRT, Danvers, MA) overnight at 4°C, followed by incubation with appropriate secondary antibodies (Proteintech, Deansgate Manchester, UK). Detection was performed using the Pierce ECL Plus Western Blotting Substrate (Thermo Scientific) according to the manufacturer's instructions.

### 2.8 RNA Isolation and Quantitative Reverse Transcription Polymerase Chain Reaction (RT‐qPCR)

Total RNA was isolated from HaCaT cells or mice epidermis using Direct‐zol RNA Microprep (Zymo Research, Irvine, CA), and cDNA was synthesized using ReverTra Ace qPCR RT Master Mix (TOYOBO, Osaka, Japan). Quantitative RT‐PCR was performed using THUNDERBIRD SYBR qPCR Mix (TOYOBO). GAPDH gene expression was used as an internal control, and the relative expression levels of each gene were determined by the 2^−ΔΔCT^ method. The primer sequences used are listed in Table 1.

**TABLE 1:** Sequences of the primers used for RT‐qPCR.

Gene	Gene arrangement
Mouse *Gapdh* forward	5′‐AGGTCGGTGTGAACGGATTTG‐3′
Mouse *Gapdh* reverse	5′‐TGTAGACCATGTAGTTGAGGTCA‐3′
Mouse *Egf* forward	5′‐GACTGGATTGGCCGGAGAA‐3′
Mouse *Egf* reverse	5′‐CGCTCCCTCCAACAACAGA‐3′
Mouse *Nav1* forward	5′‐CAGCGGTAAGCGATGATGG‐3′
Mouse *Nav1* reverse	5′‐AGCACGGTAACCACAAGCTC‐3′
Human *GAPDH* forward	5′‐CTGGGCTACACTGAGCACC‐3′
Human *GAPDH* reverse	5′‐AAGTGGTCGTTGAGGGCAATG‐3′
Human *OCLN* forward	5′‐ACAAGCGGTTTTATCCAGAGTC‐3′
Human *OCLN* reverse	5′‐GTCATCCACAGGCGAAGTTAAT‐3′
Human *FLG* forward	5′‐GGACAGGAACAATCATCGGGG‐3′
Human *FLG* reverse	5′‐CAACCTCTCGGAGTCGTCTG‐3′
Human *KRT10* forward	5′‐CCCAACTGGCCTTGAAACAA‐3′
Human *KRT10* reverse	5′‐CTGCACACAGTAGCGACCTTCT‐3′
Human *INV* forward	5′‐CCCATCAGGAGCAAATGAAC‐3′
Human *INV* reverse	5′‐GCTCGACAGGCACCTTCTG‐3′
Human *NAV1* forward	5′‐AAATGGGCGCAAGACTAGCTT‐3′
Human *NAV1* reverse	5′‐ACTGGATGTTAGAACGGGCTC‐3′

Abbreviations: *FLG*, human filaggrin; *INV*, human involucrin; *KRT10*, human keratin 10; *OCLN*, human occludin.

### 2.9 RNA Interference With NAV1

Regents for siRNA against NAV1 or non‐target control siRNA were obtained from Invitrogen (Carlsbad, CA, USA). All transfections were performed using Lipofectamine RNAiMAX Transfection Reagent (Thermo Fisher Scientific) according to the manufacturer's instructions. Cultured HaCaT cells were incubated with siRNA reagents and differentiated, followed by stimulation with EGF and 70 kDa FITC‐dextran for macropinocytosis assessment or by RNA extraction for subsequent RNA‐sequencing (RNA‐seq) analysis.

### 2.10 RNA‐Sequencing and Data Analysis

RNA‐seq datasets were generated from HaCaT cells transfected with either siRNA against NAV1 or a control siRNA. Total RNA was extracted with a Direct‐zol RNA kit (ZymoResearch). cDNA libraries for RNA‐seq were prepared using the NEBNext Ultra II Directional RNA Library Prep Kit for Illumina (New England BioLabs). All libraries were quantified using the Agilent 4200 TapeStation system (Agilent Technologies, Santa Clara, CA). Sequencing was performed on a NovaSeq 6000 system with paired‐end reads. Read alignment was conducted with the STAR genome alignment algorithm [21]. Read normalization and identification of differentially expressed genes (DEGs) were performed using HOMER with the implementation of DESeq2 [22]. Metascape was conducted for Gene Ontology (GO) and pathway analysis [23].

### 2.11 Statistical Analysis

Statistical analyses were performed using GraphPad Prism version 9 (GraphPad Software Inc., San Diego, CA). Normality of the data was assessed using the Shapiro–Wilk test. For two‐group comparisons, Student's *t*‐test was applied when homoscedasticity was confirmed, whereas Welch's *t*‐test was applied in cases of unequal variance. One‐way analysis of variance (ANOVA) followed by the Tukey post hoc test was used for multiple‐group comparisons. Statistical significance was defined as *p* < 0.05. Significant differences are illustrated as follows: *p < 0.05, **p < 0.01, ***p < 0.001, and ****p < 0.0001.

## 3 Results

### 3.1 EGF, But Not IL‐4 and IL‐13, Promotes the Uptake of 70 kDa Dextran and Internalization of Occludin in Differentiated HaCaT Cells

**FIGURE 1:**
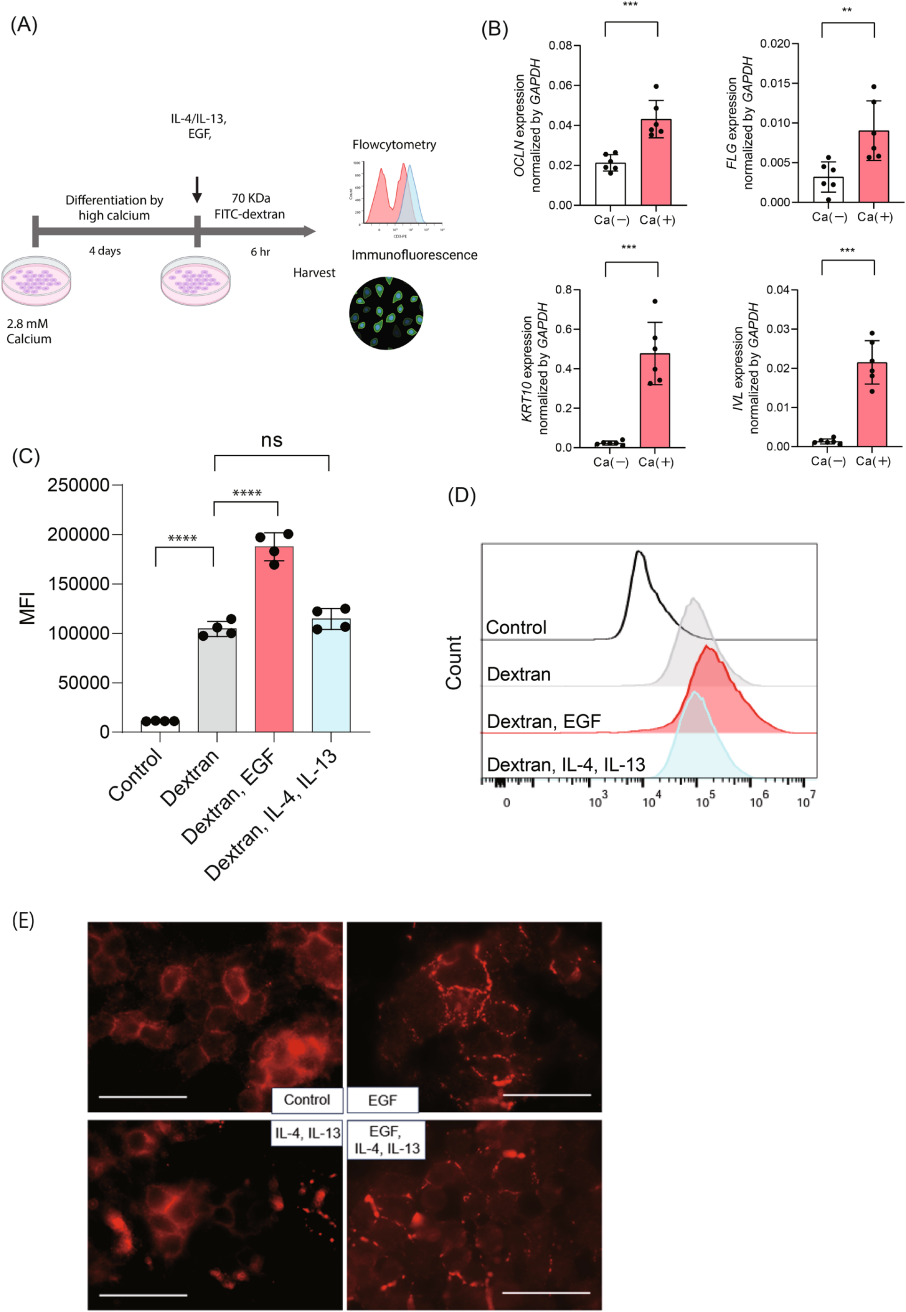
Internalization of 70 kDa FITC‐dextran is induced by EGF but not by IL‐4 and IL‐13. (A) Workflow for the assessment of macropinocytosis in HaCaT cells. HaCaT cells were seeded and differentiated in high concentrations of calcium chloride solution (2.8 mM). Cells were then stimulated with EGF (10 ng/mL), IL‐4 (100 ng/mL), and IL‐13 (100 ng/mL) or left untreated. To assess macropinocytosis, 70 kDa FITC‐dextran was added, and uptake was quantified using flow cytometry. Additionally, immunofluorescence was performed to evaluate occludin localization. (B) Gene expression levels of occludin (*OCLN*), filaggrin (*FLG*), keratin 10 (*KRT10*), and involucrin (*INV*) in HaCaT cells cultured with 2.8 mM calcium chloride for 4 days or without calcium. Data are presented as mean ± SD. Student's *t*‐test was conducted for comparison. **p < 0.01, ***p < 0.001. (C) Quantification of 70 kDa FITC‐dextran internalization by flow cytometry. Mean fluorescence intensity (MFI) was measured to assess 70 kDa FITC‐dextran uptake. Data are presented as mean ± SD. One‐way ANOVA analysis of variance with Dunnett's multiple comparisons was conducted for comparison. ****p < 0.0001. ns, not significant. (D) Representative flow cytometry histogram plots depicting the uptake of 70 kDa FITC‐dextran by HaCaT cells stimulated by IL‐4/IL‐13 or EGF. (E) Immunofluorescence images for occludin in HaCaT cells stimulated by IL‐4/IL‐13 or EGF. The scale bar represents 50 μm.

First, we investigated whether the internalization of barrier‐related proteins occurs via macropinocytosis in differentiated HaCaT keratinocytes and identified the factors that promote this process. HaCaT cells, an immortalized human keratinocyte cell line, were cultured and differentiated in high calcium concentrations (2.8 mM) to induce barrier proteins on the cell surface, after which the uptake of 70 kDa FITC‐dextran was evaluated (Figure 1A). FITC‐dextran of 70 kDa is commonly used as a marker for macropinocytosis, as molecules of this size are excluded by other endocytic pathways, including clathrin‐mediated endocytosis, and are preferentially internalized via macropinocytosis [24]. To investigate the effects of high‐concentration calcium stimulation on the differentiation of HaCaT cells, we examined the expression of differentiation‐related markers (OCLN, FLG, KRT10, and INV) using RT‐qPCR on the fourth day after calcium addition. The results demonstrated significantly higher expression levels of these markers in the calcium‐treated group compared to the untreated group (Figure 1B, OCLN: calcium‐treated group 0.043 ± 0.0085 vs. untreated group 0.021 ± 0.0037, *p* = 0.00040; KRT10: calcium‐treated group 0.48 ± 0.14 vs. untreated group 0.023 ± 0.0094, *p* = 0.00090; IVL: calcium‐treated group 0.022 ± 0.0050 vs. untreated group 0.0014 ± 0.00058, *p* = 0.0003; FLG: calcium‐treated group 0.0090 ± 0.0034 vs. untreated group 0.0032 ± 0.0017, *p* = 0.0068). These findings confirm that calcium addition successfully induces differentiation in HaCaT cells under our experimental conditions. Given that type 2 inflammation is known to impair skin barrier function [3], we examined whether stimulation with IL‐4 (100 ng/mL) and IL‐13 (100 ng/mL) promotes the uptake of 70 kDa FITC‐dextran. Additionally, we evaluated whether EGF (10 ng/mL), previously reported to enhance macropinocytosis in intestinal epithelial cells [9], contributes to this uptake in differentiated HaCaT cells. Flow cytometry analysis revealed a significant increase in 70 kDa FITC‐dextran uptake upon EGF (10 ng/mL) stimulation, while IL‐4 (100 ng/mL) and IL‐13 (100 ng/mL) had no significant effect (Figure 1C,D, Control 11 204 ± 278 vs. Dextran 104 412 ± 6528 vs. Dextran with EGF 187 586 ± 12 331 vs. Dextran with IL‐4/IL‐13 114 445 ± 9214, *p* < 0.0001 in Dextran vs. Dextran with EGF, *p* = 0.48 in Dextran vs. Dextran with IL‐4/IL‐13). EGF‐induced internalization of 70 kDa FITC dextran was also observed under undifferentiated HaCaT cells (Figure S1A,B, undifferentiated: Control 16 341 ± 544 vs. Dextran 222 000 ± 5409 vs. Dextran with EGF 525 750 ± 49 378, *p* < 0.0001 in Dextran vs. Dextran with EGF; differentiated: Control 9774 ± 195 vs. Dextran 104 915 ± 3573 vs. Dextran with EGF 197 597 ± 7009, *p* < 0.0001 in Dextran vs. Dextran with EGF), suggesting that EGF‐induced macropinocytosis occurs independently of keratinocyte differentiation status. Furthermore, to investigate whether the induction of internalization also occurs with other EGFR ligands, we examined the uptake of dextran following TGF‐α (10 ng/mL) stimulation. However, unlike EGF, TGF‐α did not significantly enhance dextran internalization (Figure S2A,B, Control 9434 ± 508 vs. Dextran 69 379 ± 6751 vs. Dextran with EGF 179 125 ± 10 472 vs. Dextran with TGF‐α 85 548 ± 8965, *p* = 0.060 in Dextran vs. Dextran with TGF‐α), suggesting that EGF‐induced macropinocytosis is ligand‐specific. Occludin, a tight junction protein with a molecular weight of 65 kDa, falls within the size range of proteins that are potentially internalized through macropinocytosis. To examine the localization of occludin, immunofluorescence staining was performed following stimulation with EGF (10 ng/mL) or IL‐4 (100 ng/mL) and IL‐13 (100 ng/mL). The results demonstrated that in differentiated HaCaT cells, occludin was localized along the cell membrane in a linear pattern. However, upon EGF stimulation, the occludin staining pattern became granular and less distinct (Figure 1E). Stimulation with IL‐4 and IL‐13 did not induce significant changes in occludin localization. These findings indicate that EGF promotes the uptake of proteins approximately 70 kDa in size, including the internalization of occludin, whereas IL‐4 and IL‐13 have no significant effect.

### 3.2 EGF‐Induced Uptake of 70 kDa Dextran and Internalization of Occludin in Differentiated HaCaT Cells Is Dependent on Macropinocytosis

**FIGURE 2:**
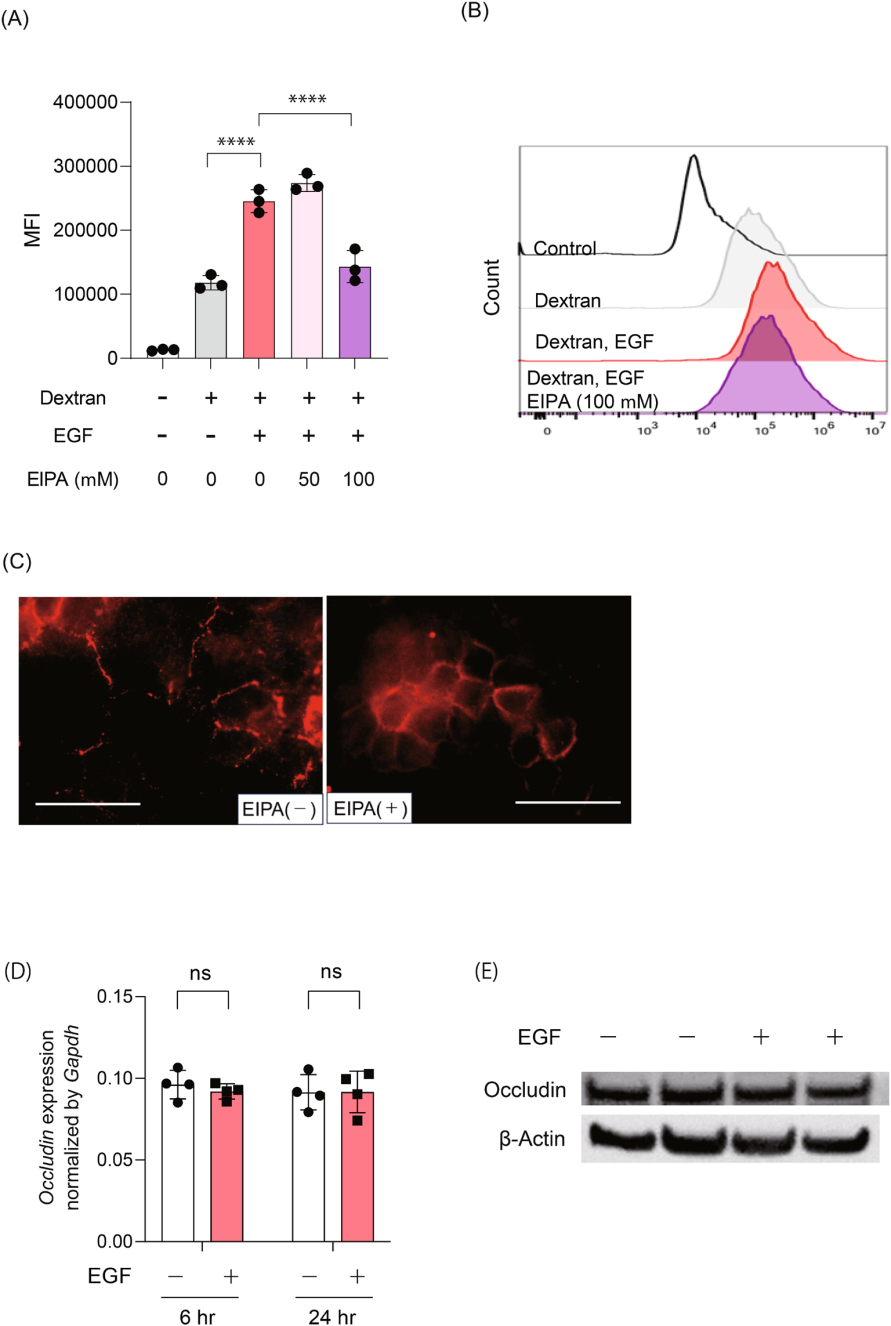
Enhancement of 70 kDa FITC‐dextran uptake by EGF is dependent on macropinocytosis. Differentiated HaCaT cells were pretreated with either 50 or 100 μM of N‐(ethyl‐N‐isopropyl)‐amiloride (EIPA) for 1 h, followed by stimulation with EGF (10 ng/mL) and addition of 70 kDa FITC‐dextran (0.5 mg/mL). (A) Quantification of 70 kDa FITC‐dextran internalization by flow cytometry. (MFI) values are shown. Data are presented as mean ± SD. One‐way ANOVA analysis of variance with Dunnett's multiple comparisons was conducted for comparison. ****p < 0.0001. (B) Representative flow cytometry histogram plots depicting uptake of 70 kDa FITC‐dextran by HaCaT cells pretreated with indicated concentrations of EIPA (50 or 100 μM), followed by EGF stimulation. (C) Immunofluorescence images for occludin in HaCaT cells pretreated with or without EIPA, followed by EGF stimulation (10 ng/mL). The scale bar represents 50 μm. (D) Gene expression levels of *OCLN* in HaCaT cells at 6 and 24 h post‐EGF stimulation. Data are presented as mean ± SD. Student's *t*‐test was conducted for comparison. (E) Immunoblotting for occludin and β‐actin in HaCaT cells at 24 h after EGF stimulation (10 ng/mL). Data are presented as mean ± SD. Student's *t*‐test was conducted for comparison. ns: Not significant.

To determine whether the internalization of 70 kDa FITC‐dextran and occludin induced by EGF is mediated via macropinocytosis, we utilized the macropinocytosis inhibitor EIPA. EIPA is widely used to distinguish macropinocytosis from other endocytic pathways, such as clathrin‐mediated endocytosis. Pre‐treatment with 100 μM EIPA significantly reduced the EGF‐induced uptake of 70 kDa FITC‐dextran (Figure 2A,B, Control 13 069 ± 1050 vs. Dextran 118 110 ± 9143 vs. Dextran, EGF 245 530 ± 28 808 vs. Dextran, 50 μM EIPA 273 816 ± 54 136 vs. Dextran, 100 μM EIPA 143 291 ± 80 173, *p* < 0.0001 in Dextran vs. Dextran with EGF, *p* < 0.0001 in Dextran with EGF vs. Dextran with EGF and EIPA). Similarly, immunofluorescence analysis showed that the EGF‐induced internalization of occludin was inhibited by pre‐treatment with EIPA (Figure 2C). Given the possibility that changes in occludin expression levels might influence its internalization upon EGF stimulation, we evaluated the expression levels of occludin in HaCaT cells. The results demonstrated no significant change in occludin expression, with or without EGF stimulation (10 ng/mL), at both 6 and 24 h (Figure 2D, 6 h: EGF‐treated group 0.092 ± 0.0040 vs. untreated group 0.096 ± 0.0076, *p* = 0.23; 24 h: EGF‐treated group 0.092 ± 0.011 vs. untreated group 0.091 ± 0.0094, *p* = 0.98). Western blotting further confirmed that the occludin protein levels remained unchanged in HaCaT cells 24 h post‐EGF stimulation (Figure 2E). These results suggest that both the uptake of 70 kDa dextran and the internalization of occludin induced by EGF are regulated by the process of macropinocytosis.

**FIGURE 3:**
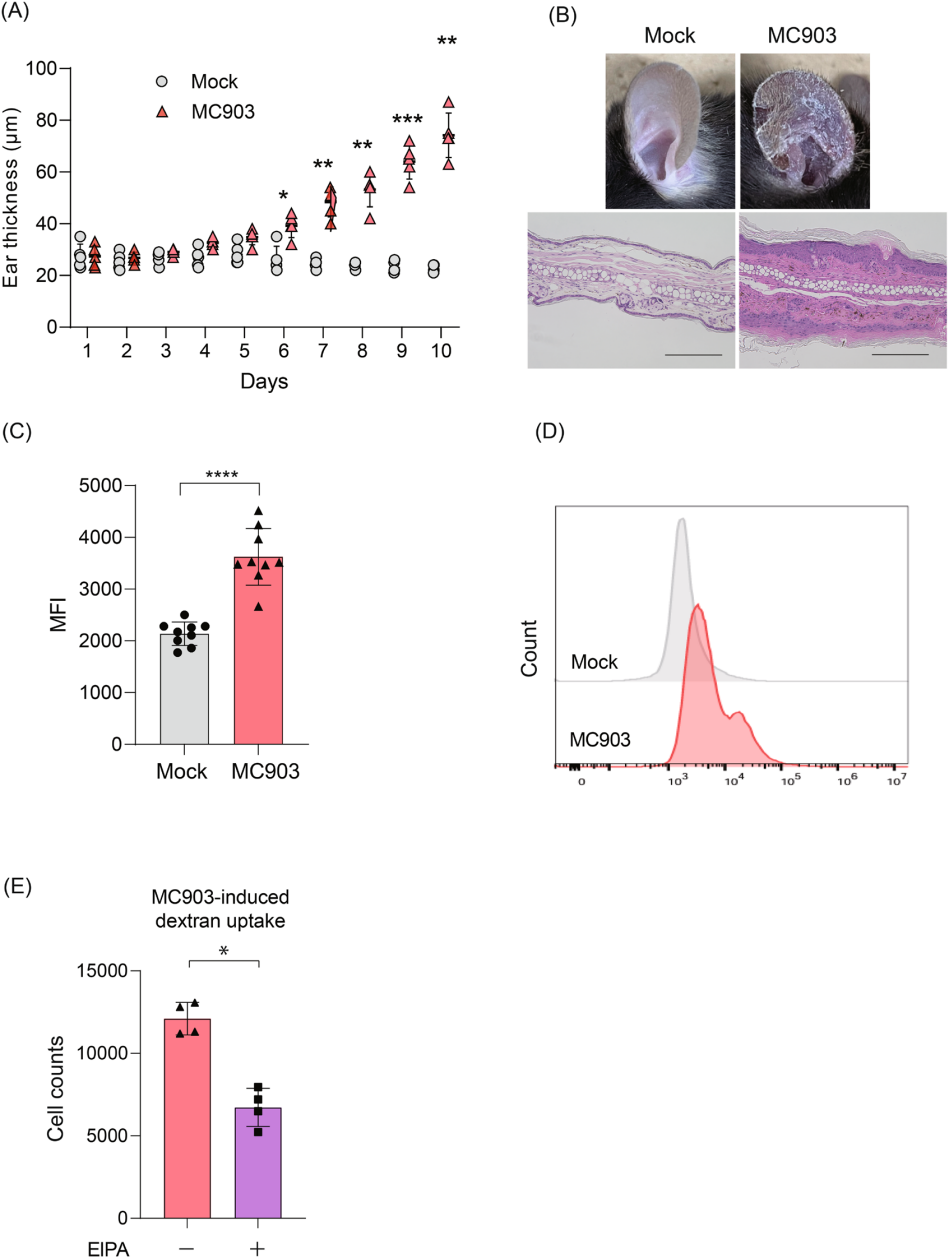
Macropinocytosis is enhanced in the epidermis of the MC903‐induced AD‐like skin dermatitis model. WT mice had either MC903 (2 nM) or vehicle applied externally to their ears for 9 consecutive days. On Day 10, 70 kDa FITC‐dextran (5 mg/mL) was applied to their ears for 1 h to evaluate macropinocytosis. (A) Changes in ear thickness of mock‐treated mice (Mock; *N* = 5) and MC903‐treated mice (MC903; *N* = 5) at the indicated time points are shown (right‐hand). Data are presented as mean ± SD. Comparison was conducted using two‐way analysis of variance, followed by Bonferroni's multiple comparisons test. *p < 0.05, **p < 0.01, ***p < 0.001. (B) Representative gross appearance and H&E staining of ear sections (left‐hand) from mice treated with mock or MC903 at Day 10. The scale bar represents 200 μm. (C) Quantification of 70 kDa FITC‐dextran uptake by flow cytometry in the epidermis of mock‐treated mice (*N* = 9) and MC903‐treated mice (*N* = 9) on Day 10. Five mice were assigned to the control group and to the MC903 application group, respectively. A total of nine ear samples were analyzed, and the results from three independent experiments were combined for analysis. Data are presented as mean ± SD. Student's *t*‐test was conducted for comparison for MFI analysis. ****p < 0.0001. (D) Representative flow cytometry histogram plots depicting 70 kDa FITC‐dextran uptake in the auricular epidermis of mock or MC903‐treated mice on Day 10. (E) Application of EIPA (10 mM) was performed prior to topical application of MC903 on Days 5–9. The number of cells showing increased uptake of 70 kDa FITC‐dextran following topical application of MC903, with (*N* = 4) or without (*N* = 4) EIPA, was assessed by flow cytometry on Day 10. Four ears from two mice were used in this experiment, and the results were analyzed in a single experiment. Data are presented as mean ± SD. Student's *t*‐test was conducted for comparison. *p < 0.05.

AD mouse models were generated by daily topical application of calcipotriol (MC903) to the ear lobes. A marked increase in ear thickness was observed in the MC903‐treated group from Day 6 compared to the control group, with a statistically significant difference (Figure 3A, Day 10 Mock 22.6 ± 1.3 vs. MC903‐treated group 74.2 ± 7.7, *p* = 0.0014). Histological analysis further confirmed increased epidermal thickness in these mice (Figure 3B). Internalization of 70 kDa FITC‐dextran in the ear epidermis was assessed by flow cytometry, revealing a significant enhancement of internalization in the MC903‐treated epidermis compared to its counterpart (Figure 3C,D, Day 10 Mock 2138 ± 215 vs. MC903‐treated group 3626 ± 517, *p* < 0.0001). To confirm that the enhanced internalization of 70 kDa FITC‐dextran was mediated by macropinocytosis, mice were pretreated with EIPA (10 mM) prior to daily MC903 application from Day 5 to Day 9. Flow cytometric analysis demonstrated that EIPA pre‐treatment significantly reduced the number of cells internalizing 70 kDa FITC‐dextran induced by MC903 (Figure 3E, EIPA‐treated group 6723 ± 1002 vs. untreated group 12 106 ± 852, *p* = 0.0004). These findings indicate that macropinocytosis is upregulated in the epidermis of AD mouse models, suggesting that the inflammatory microenvironment of AD in vivo promotes macropinocytosis.

### 3.3 EGF‐Induced 70 kDa Dextran Uptake Is Partially Mediated by NAV1

**FIGURE 4:**
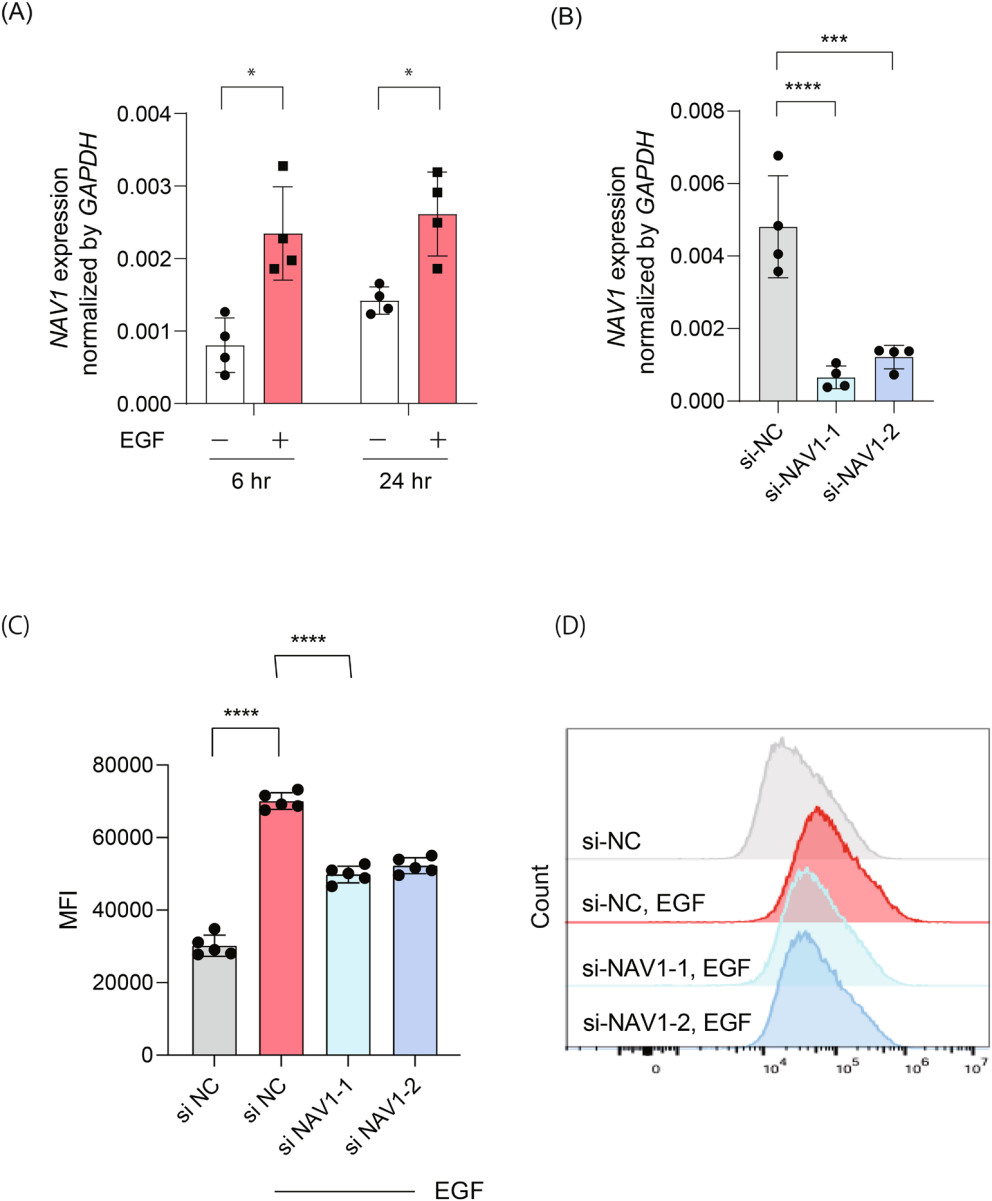
EGF‐induced macropinocytosis is partially dependent on NAV1. (A) Gene expression levels of *NAV1* in HaCaT cells at 6‐ and 24‐h post‐stimulation by EGF (10 ng/mL). Data are presented as mean ± SD. Student's *t*‐test was conducted for comparison. *p < 0.05. (B) HaCaT cells were transfected with siRNA for NAV1 (si NAV1‐1 and si NAV1‐2) or negative control siRNA (si NC). The efficiency of siRNA‐mediated gene knockdown was confirmed by RT‐qPCR. Data are presented as mean ± SD. One‐way ANOVA analysis of variance with Dunnett's multiple comparisons was conducted for comparison. ***p < 0.001. (C) HaCat cells transfected with siNAV1‐1, siNAV1‐2, or siNC were differentiated by 2.8 mM calcium chloride solution followed by EGF stimulation (10 ng/mL). Six hours after EGF application, 70 kDa FITC‐dextran uptake was assessed using flow cytometry. Data are presented as mean ± SD. One‐way ANOVA analysis of variance with Dunnett's multiple comparisons was conducted for MFI analysis. ****p < 0.0001. (D) Representative flow cytometry histogram plots depicting internalization of 70 kDa FITC‐dextran by HaCaT cells transfected with siNAV1‐1, siNAV1‐2, and siNC, followed by EGF stimulation.

The cytoskeletal protein Neuron Navigator 1 (NAV1) has been recently implicated in the enhancement of macropinocytosis and neurite outgrowth [25, 26]. Given these roles, we sought to explore whether NAV1 contributes to EGF‐mediated macropinocytosis in keratinocytes. We first assessed the impact of EGF stimulation on NAV1 expression in HaCaT cells, observing a significant upregulation of NAV1 mRNA levels at both 6 and 24 h following EGF treatment (Figure 4A, 6 h: EGF‐treated group 0.0023 ± 0.00056 vs. untreated group 0.00081 ± 0.00033, *p* = 0.0061; 24 h: EGF‐treated group 0.0026 ± 0.00050 vs. untreated group 0.0014 ± 0.00016, *p* = 0.0077). Subsequently, we evaluated the impact of NAV1 knockdown on EGF‐induced macropinocytosis. Gene expression analysis confirmed a substantial reduction in *NAV1* expression with si‐NAV1‐1 and si‐NAV1‐2 compared to si‐NC (Figure 4B, si‐NC 0.0048 ± 0.0012; si‐NAV1‐1 0.00066 ± 0.00027, si‐NAV1‐2 0.0012 ± 0.00028, *p* = 0.0001 in si‐NC vs. si‐NAV1‐1, *p* = 0.0004 in si‐NC vs. si‐NAV1‐2). Flow cytometry analysis was then performed, revealing that EGF‐induced 70 kDa FITC‐dextran uptake was significantly diminished following NAV1 knockdown (Figure 4C,D, si‐NC 30 147 ± 2624; EGF‐treated si‐NC 70 038 ± 2056; EGF‐treated si‐NAV1‐149832 ± 2058, EGF‐treated si‐NAV1‐252242 ± 1977, *p* < 0.0001 in si‐NC vs. EGF‐treated si‐NC, *p* < 0.0001 in EGF‐treated si‐NC vs. EGF‐treated si‐NAV1‐1, *p* < 0.0001 in EGF‐treated si‐NC vs. EGF‐treated si‐NAV1‐2). These findings indicate that NAV1 plays a crucial yet partial role in facilitating EGF‐mediated uptake.

### 3.4 Impact of NAV1 Depletion on Transcriptional Programs Associated With Macropinocytosis

**FIGURE 5:**
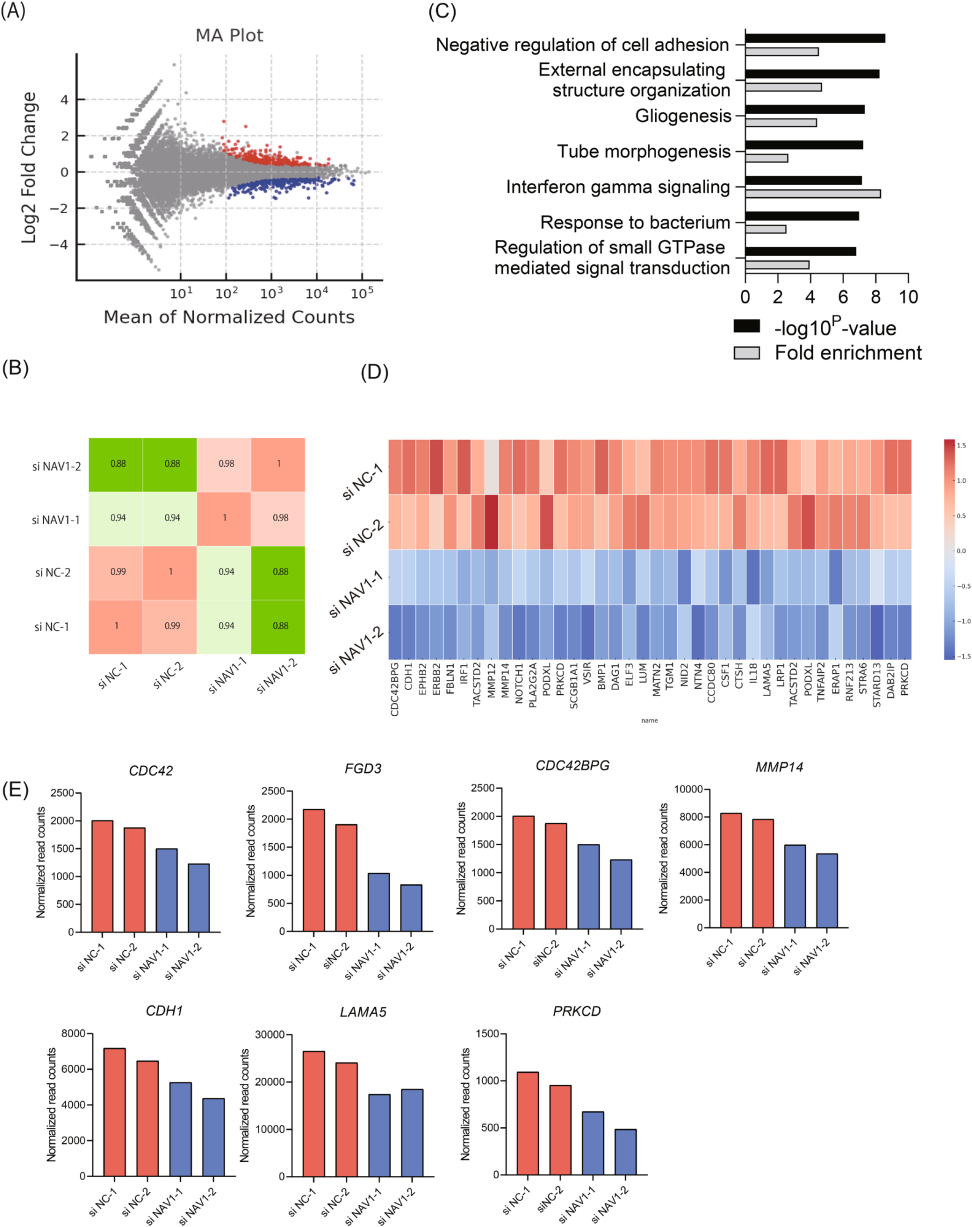
Transcriptional analysis of NAV1‐dependent signaling in HaCaT cells. (A) MA plot showing log_2_ fold changes against the mean of normalized read counts for genes of siNAV1 versus siNC. Genes with an adjusted *p*‐value < 0.1 and a positive fold change are shown in red, while those with a negative fold change are shown in blue. Representative genes are labeled. (B) Correlation matrix of RNA‐seq samples. The correlation matrix shows Pearson correlation coefficients between replicates of control (siNC‐1, siNC‐2) and NAV1 knockdown (siNAV1‐1, siNAV1‐2) samples. (C) GO and pathway analysis of down‐regulated genes in NAV1 knockdown samples. The top 7 GO terms and pathways, along with their associated −log_10_ *p* values and gene ratio, identified using Metascape, are presented. (D) Heatmap showing *Z*‐scores of normalized read counts for selected macropinocytosis‐related genes across two conditions (siNC and siNAV1) with duplicate samples for each condition. *Z*‐scores were calculated for each gene to standardize expression levels across samples. Color intensity corresponds to the degree of deviation from the mean expression level, with a gradient from blue (low) to red (high). (E) Bar graphs showing normalized read counts of representative genes associated with macropinocytosis that were downregulated in NAV1‐knockdown cells.

Based on our findings indicating NAV1's involvement in EGF‐mediated macropinocytosis, we next sought to explore underlying transcriptional changes resulting from NAV1 depletion in HaCaT cells. To identify dysregulated transcriptional programs potentially linked to macropinocytic processes, we performed RNA‐seq analysis. MA plot illustrates the distribution of genes based on log_2_ fold change and mean of normalized read counts, highlighting upregulated genes in red and downregulated genes in blue of siNAV1 compared to siNC (negative control) cells (adjusted *p*‐value < 0.1) (Figure 5A). The correlation matrix of RNA‐seq samples (siNC‐1, siNC‐2, siNAV1‐1, and siNAV1‐2) is presented in Figure 5B, indicating high reproducibility among biological replicates. Gene ontology (GO) and pathway analysis of 178 downregulated genes due to NAV1 depletion included processes associated with negative regulation of cell adhesion and external encapsulating structure organization, indicating that NAV1 supports these processes (Figure 5C). A heatmap visualization of standardized expression levels (*Z*‐scores) for selected macropinocytosis‐associated genes is presented in Figure 5D. These included Rho family GTPases, such as *CDC42* and *MMP14*, and genes associated with cell adhesion dynamics, including *CDH1* and *LAMA5*. Furthermore, genes involved in the regulation of protein localization to the membrane, such as *DAG1* and *GSN*, suggest a multifaceted role for NAV1 in coordinating these cellular processes.

### 3.5 NAV1 Expression Is Elevated in the Epidermis of AD Skin

**FIGURE 6:**
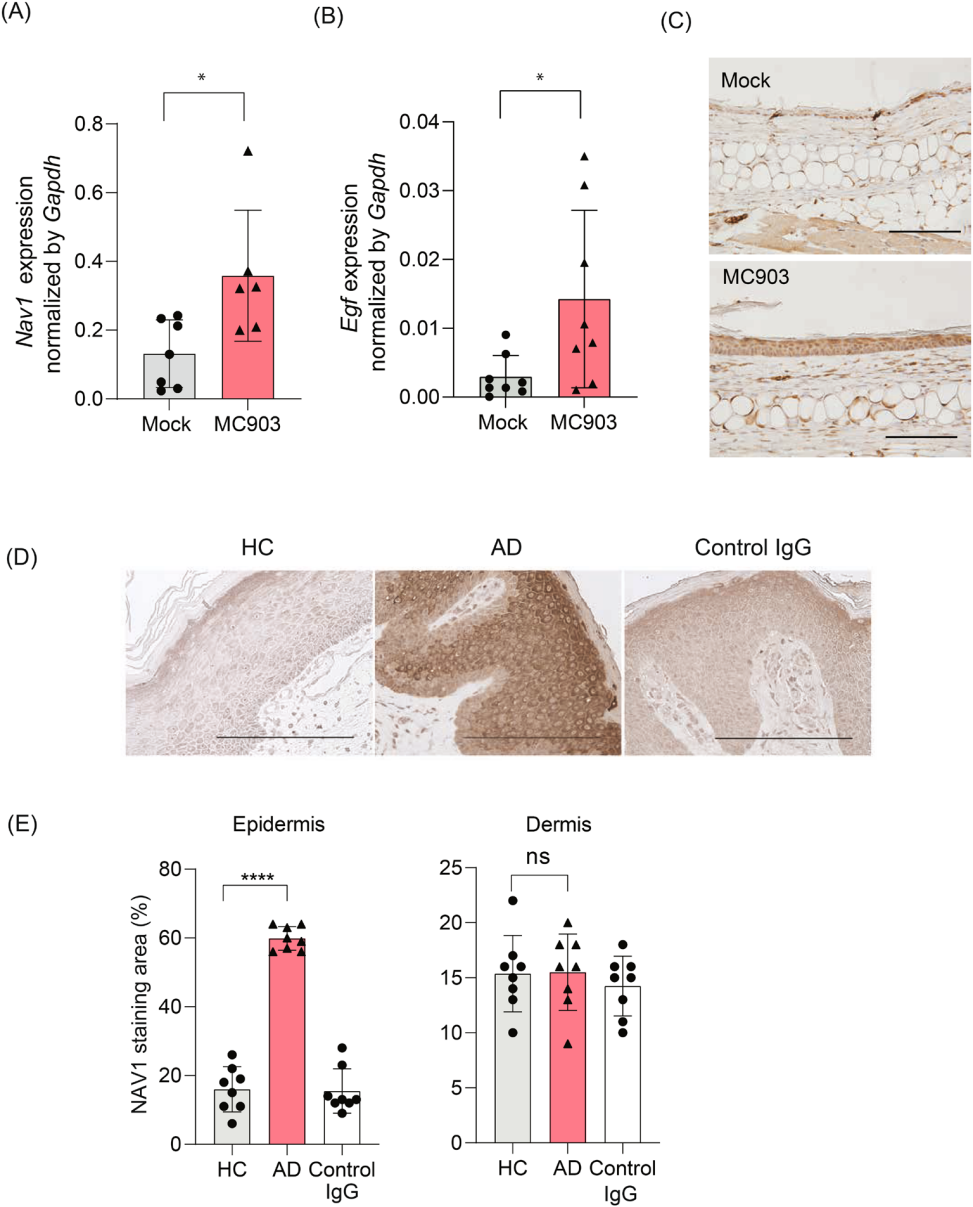
NAV1 expression and localization in the skin of the MC903‐induced AD‐like skin dermatitis model and AD patient samples. (A, B) Gene expression levels of *Nav1* (A) and *Egf* (B) in the epidermis of the ear of MC903‐treated mice (*N* = 6–8) were compared with those of the mock‐treated control group (*N* = 7–8). A total of six to eight ears from three to four mice in each group were used in this experiment, with evaluations compiled from two independent experiments. Data are presented as mean ± SD. An unpaired *t*‐test with Welch's correction was conducted for comparison. *p < 0.05. (C) Immunohistochemical staining of NAV1 in the skin of mice treated with mock or MC903 at Day 10. Scale bar is 100 μm. (D) Immunohistochemical staining of NAV1 in the skin sections from healthy controls and patients with AD. Results are representative of 4 healthy controls and 4 AD patients. The scale bar represents 200 μm. (E) The percentage of areas with NAV1 staining in the epidermis and dermis of the lesional skin of AD and the skin of healthy counterparts. Data are presented as mean ± SD. One‐way ANOVA analysis of variance with Dunnett's multiple comparisons was conducted for comparison. ****p < 0.0001. ns, not significant.

The gene expression levels of *Nav1* in the ear epidermis of six AD mouse models and seven control mice were evaluated by RT‐qPCR. Compared to the control group, *Nav1* and *Egf* gene expression levels were significantly elevated in the AD model mice (Figure 6A, Mock 0.13 ± 0.090 vs. MC903‐treated group 0.36 ± 0.17, *p* = 0.019, Figure 6B, Mock 0.0030 ± 0.0029 vs. MC903‐treated group 0.014 ± 0.012, *p* = 0.043). Elevated gene expression of EGF in the epidermis of AD mouse models may suggest that EGF contributes to the increased macropinocytosis observed in MC903‐treated skin. Immunohistochemical staining was then conducted on skin tissues from both the animal models and patient samples. NAV1 was expressed on epidermal cells of both (Figure 6C,D). The intensity of NAV1 staining in the epidermis was significantly stronger in the AD skin compared to its healthy counterpart (Figure 6E, Epidermis: HC 16 ± 6.1 vs. ad 60 ± 3.2 vs. Control IgG 16 ± 6.1, *p* < 0.0001 in HC vs. AD, *p* = 0.50 in HC vs. Control IgG). In contrast, the dermis of both AD and healthy skin showed overall weak NAV1 staining, similar to that observed with control IgG staining (Figure 6E, Dermis: HC 15 ± 3.2 vs. ad 16 ± 3.2 vs. Control IgG 14 ± 2.5, *p* = 1.0 in HC vs. AD, *p* = 0.71 in HC vs. Control IgG).

## 4 Discussion

The present study observed an increase in macropinocytosis induced by EGF, but not by inflammatory cytokines IL‐4 and IL‐13, in differentiated keratinocytes, which was accompanied by the internalization of the tight junction protein occludin. Enhanced macropinocytosis was also confirmed in the epidermis of an AD mouse model. NAV1 was identified as a novel EGF‐dependent regulator of macropinocytosis, with its expression elevated in the epidermis of both AD patients and the AD mouse model. These findings suggest that EGF‐dependent macropinocytosis may be closely linked to barrier dysfunction in skin diseases, including AD.

Macropinocytosis is involved in the process of cellular uptake of extracellular fluid and molecules through dynamic remodeling of the actin cytoskeleton [12, 27]. This process is regulated by several well‐established pathways, including the Rho family GTPase pathway, the Trio RhoGEF pathway, the Phosphoinositide 3‐Kinase (PI3K) pathway, and the Protein Kinase C (PKC) pathway [24, 28, 29, 30, 31, 32]. Previous reports have demonstrated that NAV1 interacts and colocalizes with Rho guanine nucleotide exchange factor (GEF) TRIO, leading to the activation of Rho family proteins such as Rac1 and RhoG [28]. In our comprehensive gene expression analysis, associations between NAV1 and several Rho family proteins were also observed in keratinocytes. Among them, Rac1 and Cdc42 are key regulators of actin reorganization and macropinocytosis activation [31, 33], and NAV1 knockdown resulted in a decrease in Cdc42 expression. Several upstream regulators of Rac1 and Cdc42, including Dock, FGD, and VAV family members, have been identified [34, 35], and this study has observed reduced expression of FGD3, one of these critical regulators. Additionally, downstream effectors of Rac1 and Cdc42, such as CDC42BPG, also showed decreased expression in NAV1‐knockdown cells. Furthermore, the expression of Matrix Metalloproteinase‐14 (MMP‐14), an enzyme involved in extracellular matrix remodeling and known to promote RhoA‐dependent internalization [36], was also downregulated in the NAV1‐knockdown group. These findings suggest that NAV1 is linked to macropinocytosis through multiple Rho family‐mediated pathways, supporting previous reports of NAV1's involvement in Rho family protein signaling [26, 28].

The regulation of macropinocytosis is also influenced by cell adhesion molecules [37]. Cell adhesion factors, such as Cadherin 1 (CDH1) and Laminin Subunit Alpha5 (LAMA5), regulate cell‐to‐cell and cell‐to‐extracellular matrix (ECM) interactions, playing a crucial role in controlling cell shape and motility [38, 39]. These factors are essential for the actin cytoskeleton rearrangements required for macropinocytosis. In this study, the expression of CDH1 and LAMA5 was decreased in NAV1‐knockdown keratinocytes, suggesting a potential role in facilitating macropinocytosis. Additionally, the PRKCD gene, which encodes PKCδ and is associated with the PKC pathway [40], also exhibited reduced expression in NAV1‐knockdown cells. These findings collectively indicate that NAV1 modulates macropinocytosis through a complex network of pathways.

In the AD mouse model, we observed enhanced uptake of 70 kDa dextran in the epidermis, which was partially inhibited by the macropinocytosis inhibitor EIPA, indicating that macropinocytosis is upregulated in inflammatory skin diseases in vivo. Given that EGF expression was also elevated in the epidermis of AD model mice, it is possible that EGF contributes to this process. However, EGFR activation can be induced by multiple ligands, and while some may also regulate macropinocytosis, our findings suggest that EGF is a potential contributor rather than the sole driver of this process in the AD microenvironment. The internalization of tight junction proteins, such as occludin, by EGF suggests that macropinocytosis may play a broader role in diseases beyond AD. In neoplastic diseases, EGF‐induced macropinocytosis may support cancer cell proliferation and survival by enhancing nutrient uptake. Similarly, in other inflammatory skin conditions such as psoriasis, where the expression of growth factors, including EGF, is upregulated [41, 42], similar mechanisms involving the internalization of barrier proteins may occur within the lesional skin. While our study highlights the involvement of macropinocytosis in AD and suggests a role for EGF in this process, further research is needed to clarify its relative contribution and the potential involvement of other EGFR‐activating factors.

**FIGURE 7:**
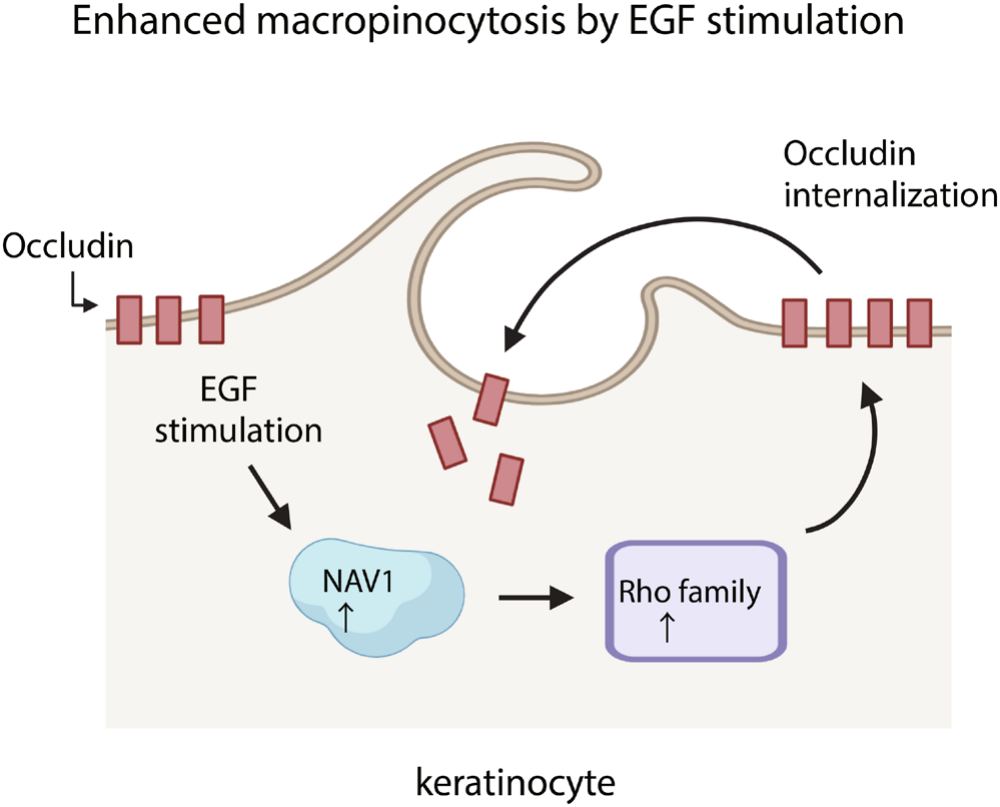
Proposed model of EGF‐induced macropinocytosis in keratinocytes. Under homeostatic conditions, keratinocytes maintain a stable barrier function with minimal macropinocytosis. Upon EGF stimulation, NAV1 and members of the Rho family are induced, leading to enhanced macropinocytosis. This process involves internalization of a tight junction protein, occludin, which may disrupt the epidermal barrier.

In summary, this study elucidated the enhancement of EGF‐ and NAV1‐dependent macropinocytosis in keratinocytes (Figure 7). The proper formation of a functional skin barrier requires not only the adequate expression of barrier‐related proteins but also their correct localization. While EGF stimulation did not alter the overall expression levels of occludin, it did enhance its internalization, thereby disrupting its proper localization. Our findings reveal a novel mechanism by which macropinocytosis regulates the skin barrier, suggesting that inhibition of macropinocytosis may prevent the internalization of barrier proteins like occludin, thereby reinforcing barrier function and potentially offering an antimicrobial benefit by blocking the entry of pathogens.

## Author Contributions

Haruka Taira and Sayaka Shibata designed the experiments. Haruka Taira and Sayaka Shibata wrote the manuscript. Haruka Taira performed most of the experiments with contributions from Lixin Li, Asumi Koyama, Kentaro Awaji, Rino Toyoshima, Toyoki Yamamoto, Yukiko Ito, Eiki Sugimoto, Yuka Mizuno, and Sayaka Shibata. Haruka Taira and Sayaka Shibata analyzed the data. Shinichi Sato supervised the project. All authors discussed the results and commented on the manuscript.

## Conflicts of Interest

The authors declare no conflicts of interest.

## Supporting information


Figure S1.

Figure S2.

## Data Availability

The RNA‐seq data that support the findings of this study will be deposited in the DDBJ Sequence Read Archive (DRA) under the accession number [XXXXXXX]. The data will be openly available upon publication.
