# Supplementary figures and images for: EGF‐Induced Macropinocytosis Promotes NAV1‐Dependent Internalization of Occludin in Keratinocytes
Source: FASEB J. 2025 Apr 23;39(8):e70564. doi: 10.1096/fj.202402876R (PMC12017258; doi:10.1096/fj.202402876R)

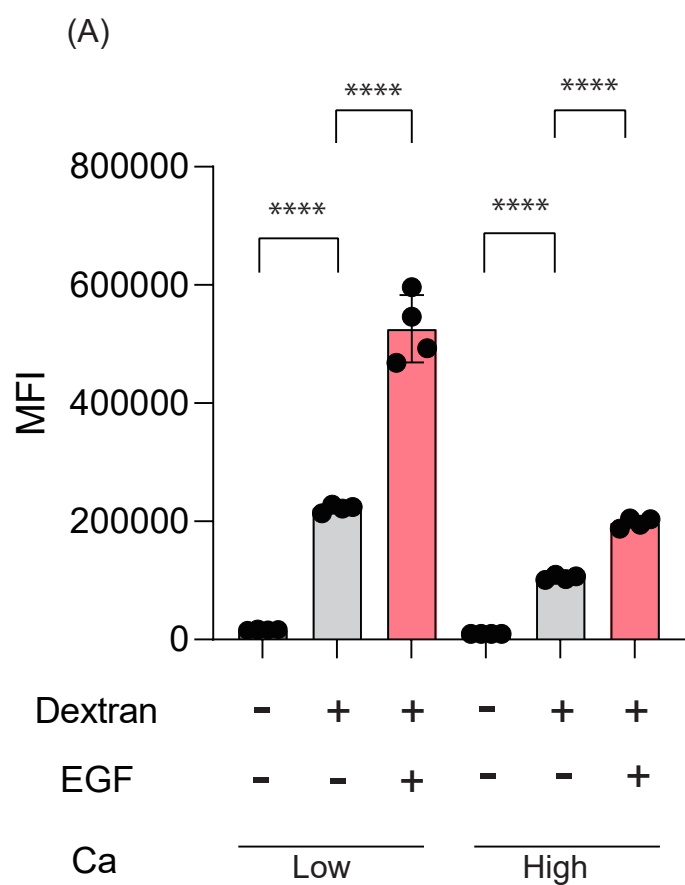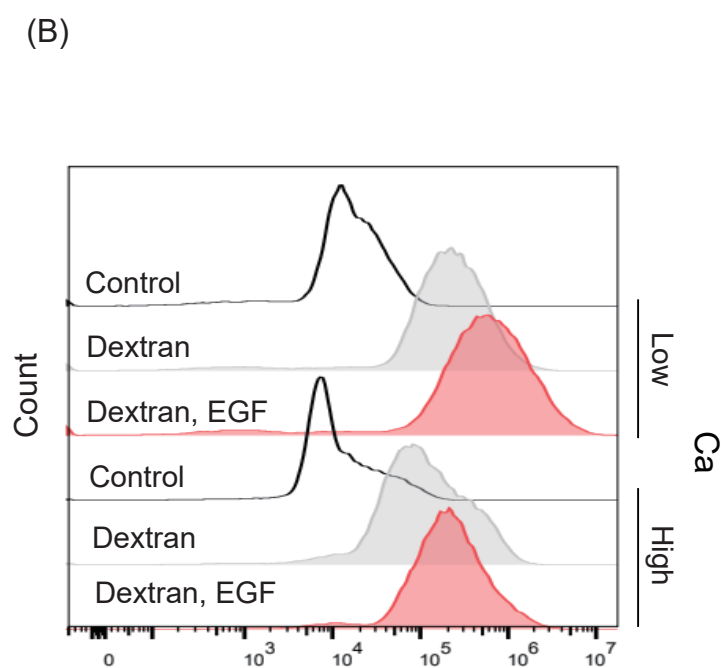

Supplement: Supplementary file 1 — Figure S1. [file FSB2-39-e70564-s001.pdf]

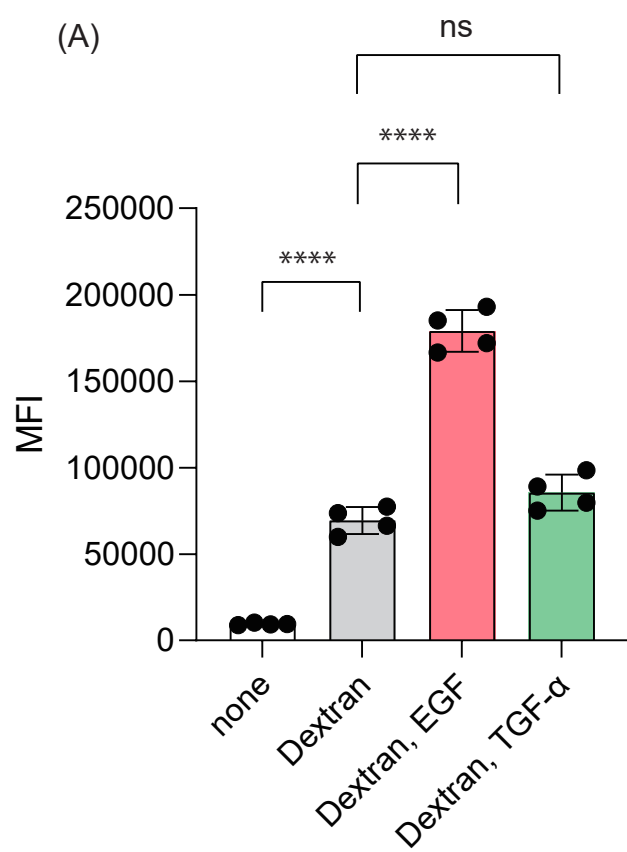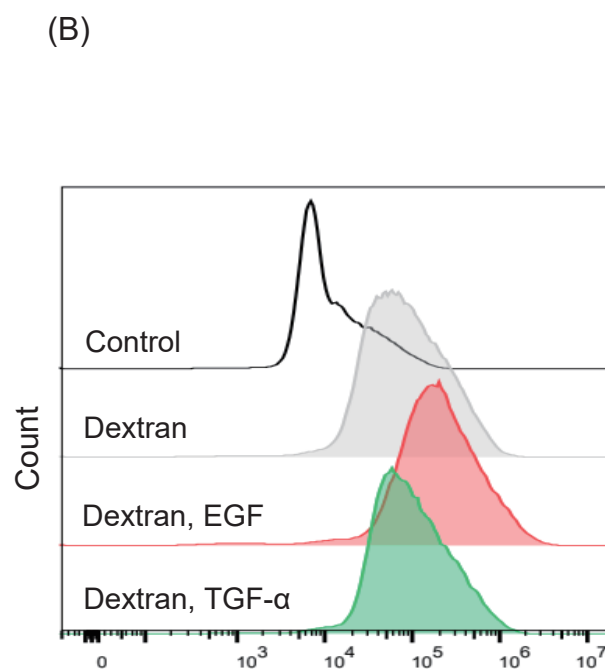

Supplement: Supplementary file 2 — Figure S2. [file FSB2-39-e70564-s002.pdf]
